# Enhancing the Inhomogeneous Photodynamics of Canonical Bacteriophytochrome

**DOI:** 10.1021/acs.jpcb.2c00131

**Published:** 2022-03-31

**Authors:** Jakub Rydzewski, Katarzyna Walczewska-Szewc, Sylwia Czach, Wieslaw Nowak, Krzysztof Kuczera

**Affiliations:** † Institute of Physics, Faculty of Physics, Astronomy and Informatics, Nicolaus Copernicus University, Grudziadzka 5, 87-100, Torun, Poland; ‡ Department of Molecular Biosciences, University of Kansas, Lawrence, Kansas 66047, United States; ¶ Department of Chemistry, University of Kansas, Lawrence, Kansas 66045, United States

## Abstract

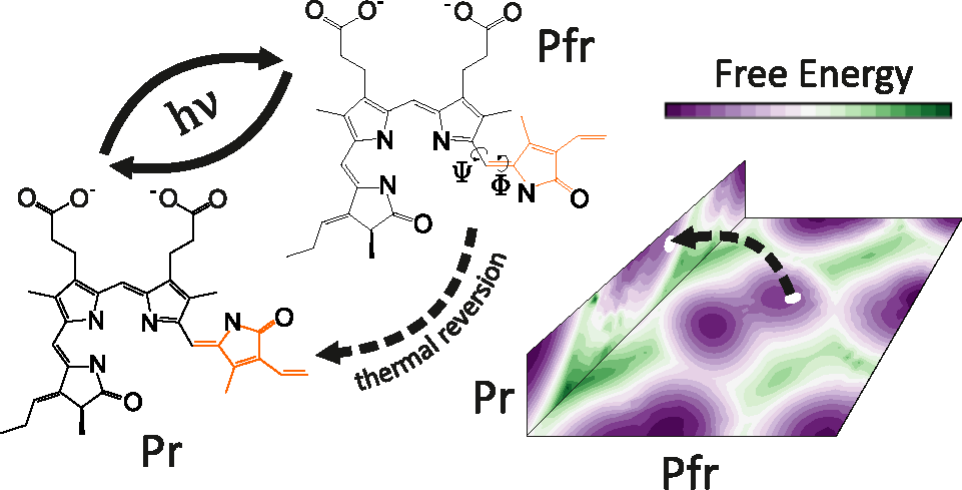

The ability of phytochromes to act as photoswitches in plants and microorganisms depends on interactions between a bilin-like chromophore and a host protein. The interconversion occurs between the spectrally distinct red (Pr) and far-red (Pfr) conformers. This conformational change is triggered by the photoisomerization of the chromophore D-ring pyrrole. In this study, as a representative example of a phytochrome-bilin system, we consider biliverdin IXα (BV) bound to bacteriophytochrome (BphP) from *Deinococcus radiodurans*. In the absence of light, we use an enhanced sampling molecular dynamics (MD) method to overcome the photoisomerization energy barrier. We find that the calculated free energy (FE) barriers between essential metastable states agree with spectroscopic results. We show that the enhanced dynamics of the BV chromophore in BphP contributes to triggering nanometer-scale conformational movements that propagate by two experimentally determined signal transduction pathways. Most importantly, we describe how the metastable states enable a thermal transition known as the dark reversion between Pfr and Pr, through a previously unknown intermediate state of Pfr. We present the heterogeneity of temperature-dependent Pfr states at the atomistic level. This work paves a way toward understanding the complete mechanism of the photoisomerization of a bilin-like chromophore in phytochromes.

## 1 Introduction

Isomerization is the hallmark of light-activated molecular switches.^1,2^ It is also one of the simplest means to convert light into mechanical motion. At the atomistic level, a series of conformational changes in light-responsive proteins, referred to as the protein photocycle, is triggered by absorbing the energy of a photon. One example of complexes in which photoisomerization can induce a large conformational change in the protein environment is the phytochrome family which represents a diverse class of photoreceptors present in plants, and fungal and bacterial kingdoms.^3,4^ The phytochrome family uses a lineage of the GAF domain that binds a thioether-linked tetrapyrrole chromophore for light sensing.^5−8^ Phytochromes play an essential role in light-regulated processes, e.g., phototaxis, pigmentation, and photosynthesis, and are interesting also as targets for optogenetic applications, imaging in tissues, light-dependent gene expression, and possible medical applications.^9−14^ BphP in the *Deinococcus radiodurans* cells, in addition to its responsibility for pigmentation and growth, is potentially involved in the resistance of these bacteria to ionizing radiation and mutagenic factors by participating in the DNA damage response.^15^

**Figure 1:**
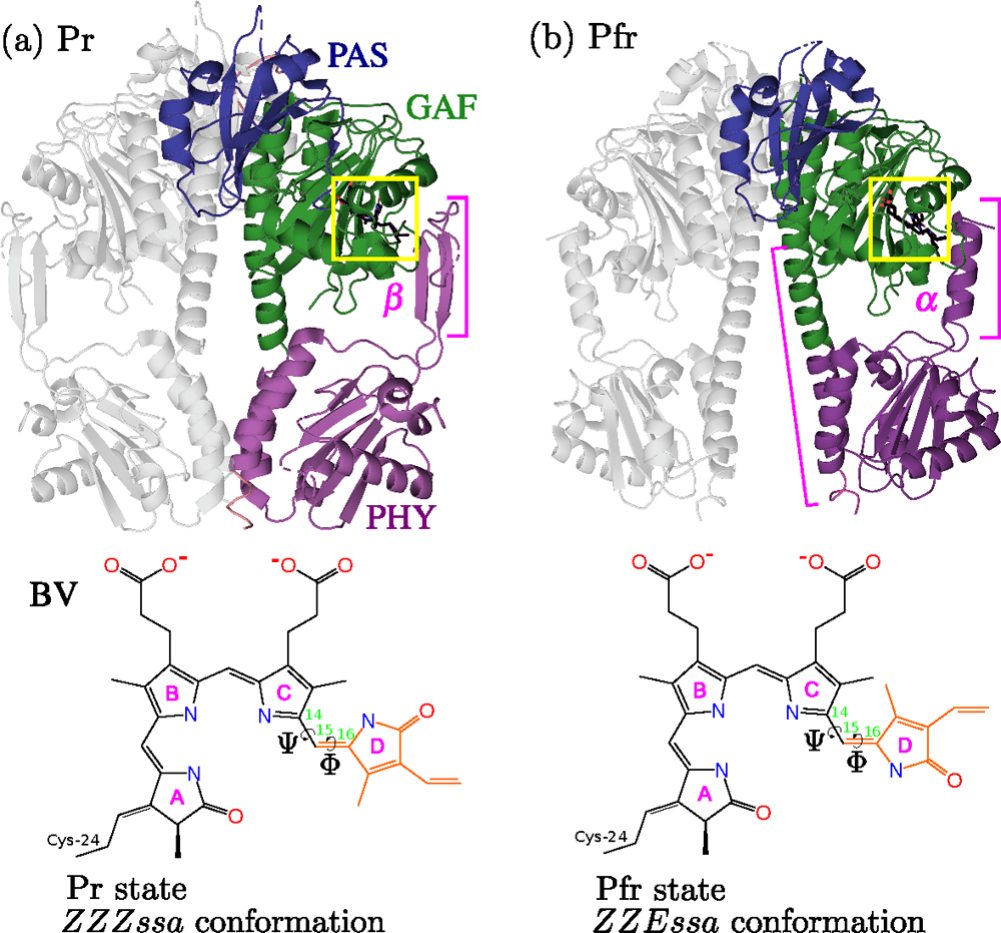
Overview of structural features of the BphP-BV complex from *D. radiodurans* in the (a) Pr state (PDB: 4O0P) and (b) Pfr state (PDB: 4O01).^17^ The first subunit is colored and labeled by its PAS, GAF, and PHY domains, whereas the second subunit is gray. The BV chromophore is enclosed in yellow boxes. The BV chromophore is presented in the *ZZZ* and *ZZE* conformations with labeled pyrrole rings (A, B, C, D), and the dihedral angles between C- and D-ring: Φ: C15=C16, Ψ: C14–C15. The tongue region is labeled by its secondary structure: β in Pr and α in Pfr. Large conformational changes propagated by the *ZZZ* to *ZZE* isomerization are secondary-structure rearrangements in the PHY-GAF helix from β-sheet (Pr) to α-helix (Pfr) and the opening of the PHY-tongue region.

Phytochromes can assume two interchangeable forms: Pr and Pfr. The core of a light-regulated switch in phytochromes is based on cycling between Pr and Pfr.^16,17^ These forms are based on their respective absorption maxima in the red and far-red spectral regions. The corresponding transition is triggered by the *ZZZ* to *ZZE* (Pr to Pfr) or the *ZZE* to *ZZZ* (Pfr to Pr) photoisomerization.^2^ Subsequently, the event leads to a secondary structure change of the flexible and partially disordered tongue region and to the straightening of the α-helix spine (Figure 1), which both alter protein–protein interactions via long-range allosteric signaling.^18^ The photosensory core module of most BphPs has a tripartite region comprising three conserved domains: PAS, GAF, and PHY.^4^ In prototypical phytochromes, the bilin chromophore embedded in the GAF domain adopts most likely a protonated configuration in the Pr state.^19^ The light absorption involves the rotation of the D-ring pyrrole in the chromophore around a double carbon bond located between C- and D-ring pyrroles (C15=C16, Figure 1). The chromophore, tetrapyrrole bilin attached via thioether linkage, resides in the chromophore-binding domain (CBD). The exact nature of the chromophore varies for different subfamilies of phytochromes; e.g., BV is the native chromophore in the proteobacterial phytochromes, and it is attached covalently to a cysteine residue via the A-ring pyrrole vinyl side chain.

The understanding of the phytochrome family has been largely driven by experimental studies employing IR,^20^ resonance Raman,^21−24^ NMR,^25^ and X-ray crystallography.^17,26−29^ In contrast to other photoactive systems such as rhodopsin and photoactive yellow protein, there are few recent computational studies attempting to determine how the signal is relayed from the chromophore to the protein matrix.^30^ However, it has been suggested that the chromophore fluctuations are more complex when a molecular switch is embedded in the protein environment.^5,31−33^ What is arguably more important, experimental results have shown a possible existence of thermally dependent states of various phytochromes,^34^ likely connected to the concept of weakly coupled equilibria.^35,36^ It is unknown how such states relate to the thermal dark reversion of Pfr to Pr at the atomistic level.^30^ Moreover, it is widely speculated that the photoisomerization is triggered mainly at the double bond between C- and D-ring (C15=C16) but it is not clear if a single-bond rotation is also possible (C14–C15).^2,37^ Overall, the structural and thermodynamical determinants of photoreception in phytochromes are still poorly understood.^38^

The goal of this article is to study, from the thermodynamical viewpoint, the early stages of the mechanical signal transduction in the BphP-BV complex and the metastable states of Pr and Pfr photoproducts through MD simulations. However, this goal poses a major difficulty due to the so-called the Boltzmann sampling problem^39^ caused mainly due to the existence of long time scales involved in the dynamics of photoproducts.^20^ To achieve such a challenging goal, we employ enhanced sampling MD simulations to overcome the FE barrier of the primary photoisomerization event and to efficiently sample nonequilibrium configurations visited by the BphP-BV complex in the Pr and Pfr conformers. By performing simulations with an aggregate time of ∼2 μs, we study the Pr and Pfr conformers starting from their equilibrium configurations, i.e., before the photoisomerization takes place (Pr) and after the primary photochemical event is concluded (Pfr). The ultrafast photochemical steps during the primary photoisomerization event (Figure S1) cannot be observed here. However, regardless of the transitions between the short-lived intermediates during the photoisomerization,^40−44^ these steps lead to either Pfr or Pr which we can study using enhanced sampling MD.

In this paper, (i) we show that Pr and Pfr conformers are characterized by distinctly different FE landscapes in which metastable states allow temperature-dependent transitions related to the dark thermal reversion. This transition proceeds through a previously unknown intermediate state of Pfr caused by the rotation of Arg-466, His-467, and Ser-468 from the tongue region. (ii) We characterize FE barriers between metastable states accompanying the Pr to Pfr transition and find they agree with spectroscopic results. (iii) We reveal the fundamental thermodynamical role of a protein environment acting as a selectivity filter for deepening and widening important metastable states in Pfr. (iv) Finally, we find that, the enhancement of the local fluctuations of the BV chromophore contributes to nanometer-scale conformational movements in BphP, following two experimentally determined signal transduction pathways. In our opinion, this work represents an important step toward understanding thermodynamical characteristics of chromophore-bound canonical phytochromes in the Pr and Pfr states.

## 2 Methods

### 2.1 Models

We perform all MD simulations using the gromacs 2018.1 code^45^ patched with the plumed 2.8 plugin.^46,47^ As the starting structures of the BphP-BV complexes (Figure 1), we use the Pr and Pfr X-ray structures: Pr (dark state; ∼660 nm red light absorption; PDB 4O0P(17)) and Pfr (illuminated state; ∼700 nm far-red light absorption; PDB 4O01(17)). The crystal structures consists of PAS-GAF-PHY domains and BV covalently attached to Cys-24. We use the AMBER03 force field.^48^ The gromacs topology for BV is taken from ref (19), where the authors have used ab initio quantum chemical calculations for the parametrization of the *ZZZ* and *ZZE* BV chromophores. The BV chromophore is modeled with all nitrogen atoms protonated as suggested in ref (19). We refer to Table S1 for further details about the protonation states of the titratable residues. We solvate the complexes with TIP3P water molecules^49^ in a box of 0.7 nm in all directions, neutralize the complexes, and use 0.15 M concentration of Na^+^ and Cl^–^ ions. All of the simulations use a 2 fs integration time step. We use LINCS^50^ to constrain bonds involving hydrogen atoms, including heavy atom to hydrogen bonds in the protein and chromophore, as well as O–H and artificial H–H bonds in the rigid TIP3P water model. We apply periodic boundary conditions. The long-range electrostatic interactions are handled using particle-mesh Ewald^51^ with a cutoff of 1.2 nm and a cutoff of 1.2 nm for the nonbonded van der Waals interactions. The complexes are minimized for 5000 steps until convergence is obtained and equilibrated using velocity rescaling thermostat^52^ at 300 K with a relaxation time of 1 ps to simulate the systems in the canonical ensemble (*NVT*). Subsequently, unbiased MD simulations are carried out for 200 ns for each protein–ligand complex to check the level of thermal fluctuations of the equilibrium metastable states.

### 2.2 Enhanced Sampling Simulations

We run enhanced sampling MD simulations using variationally enhanced sampling^53^ (VES) as implemented in the VES module available in plumed.^46,47^ The parameters used to launch the simulations remain the same as for the unbiased simulations (Section 2.1). The enhanced sampling simulations are carried out for 500 ns in the Pr and Pfr forms of BphP-BV complex, while the BV chromophores in the *ZZZ* and *ZZE* conformations are run for 200 ns. The bias in our simulations is represented as a Fourier series, *V*_**α**_(**z**) = *∑*_**k**_α_**k**_e^*i***kz**^, where **α** = [α_–*N*_, ..., α_*N*_] are real variational coefficients and **z** ≡ (Φ, Ψ) represents the set of the biased variables as depicted in Figure 1 (see Section 3.1 for a definition). We use the order of the expansion as *N* = 10, the periodic domain of the basis set as [ – π, π], and **k** ≡ [*k*_1_, *k*_2_, ... ], where each term goes from −*N* to *N*. We employ a uniform distribution as the target probability distribution, *p*_*T*_, that should be obtained once the simulations are converged. The functional Ω[*V*_**α**_] (see Section S3 for a definition) is optimized using a version of stochastic gradient descent^54^ (SGD) with a learning rate μ = 0.1 and a stride of 1000 steps. During the analysis stage, we discard the first 10 ns of the simulations to ensure that we avoid the period at the beginning of the simulations where the statistical weights, *e^βV^*, may not be equilibrated due to initial fast changes in the bias values. The remaining samples are used in the construction of FE landscapes. The weights are scaled to the range 0 to 1 to avoid numerical issues. A detailed introduction to VES can be found in Section S3.

### 2.3 Analysis and Data

All atomistic trajectories are analyzed using gromacs 2018.1^45^ and plumed 2.8.^46,47^ The data supporting the results of this study are openly available from plumed nest(47) under plumID:22.015 at https://plumed-nest.org/eggs/22/015/.

## 3 Results

### 3.1 Collective Variables for Enhanced Chromophore Dynamics

We start with the preparation of the structures of BphP from *D. radiodurans* with the BV chromophore in the Pr (PDB: 4O0P) and Pfr (PDB: 4O01) conformers,^17^ sometimes referred to as the dark and illuminated forms, respectively. Both crystallographic structures comprise the core photosensory module (PAS, GAF, and PHY) and the BV chromophore covalently attached to the side chain of Cys-24 via a thioether linkage with the A-ring pyrrole (Figure 1). The complexes are prepared for MD simulations in four variants: the BphP-BV complex in the Pr and Pfr forms and the solvated BV in the *ZZZ* and *ZZE* forms, i.e., without the protein environment. The BV chromophore is modeled with all nitrogen atoms protonated as it has been recently found that the BV chromophore in a fully protonated form agrees with absorption spectra.^19^ For a detailed protocol regarding the preparation of the systems for simulations, we refer to Section 2.1.

As we want to study how the mechanical signal is propagated through the protein environment in the Pr and Pfr conformers, we only enhance the chromophore dynamics. To this aim, we start by selecting degrees of freedom suitable for the chromophore embedded in the CBD region of BphP. For this, we construct a set of generalized variables for a quantitative description of the chromophore dynamics in the CBD region of BphP. These generalized variables are typically referred to as collective variables (CVs). Upon the transition from *ZZE* to *ZZZ*, the main change in the BV chromophore occurs between C- and D-ring pyrroles (Figure 1). Thus, as a first CV, we take the Φ dihedral angle (C15=C16, Figure 1) involving the rotation of the D-ring pyrrole around double carbon bond.^4,17,55^ As a second CV, we select the Ψ dihedral angle describing the rotation of the D-ring pyrrole around single carbon bond (C14–C15, Figure 1b) because of a possible involvement in the chromophore photoisomerization.^26,56^

After selecting the CVs, we run 200 ns of unbiased MD simulations at 300 K to check whether the systems are stable in the Pr and Pfr forms and observe the magnitude of the equilibrium fluctuations of the CVs in the X-ray conformations of Pr and Pfr. We find that during 200 ns the thermal fluctuations of Pr and Pfr do not allow for the sampling of other metastable states. The systems are kinetically restricted to the basins of the X-ray structures (Figure S2). Next, we move to enhanced sampling simulations that are run at 300 K for 500 ns for both conformers of the protein–chromophore complex and 200 ns simulations for the solvated chromophore. For further details regarding parameters used to run the unbiased MD and enhanced sampling simulations, see Sections 2.1 and 2.2, respectively.

### 3.2 Thermodynamical Landscapes from Variational Sampling

To drive the X-ray structures out of equilibrium and reconstruct FE landscapes, we choose an enhanced sampling MD approach known as variationally enhanced sampling (VES).^53^ In VES, a convex functional of the biasing potential, Ω[*V*(**z**)], is minimized during the simulation to estimate FE, *F*(**z**). The biasing potential *V*(**z**) acts in the CV space and is represented by a selected combination of basis functions. At convergence, FE can be estimated using umbrella reweighting.^57^ As such, VES is an efficient method for exploring long-time scale events.^53,58^ For conciseness, the theory behind CV-based enhanced sampling and a detailed discussion of the method used for the simulations are provided in Section S3.

**Figure 2:**
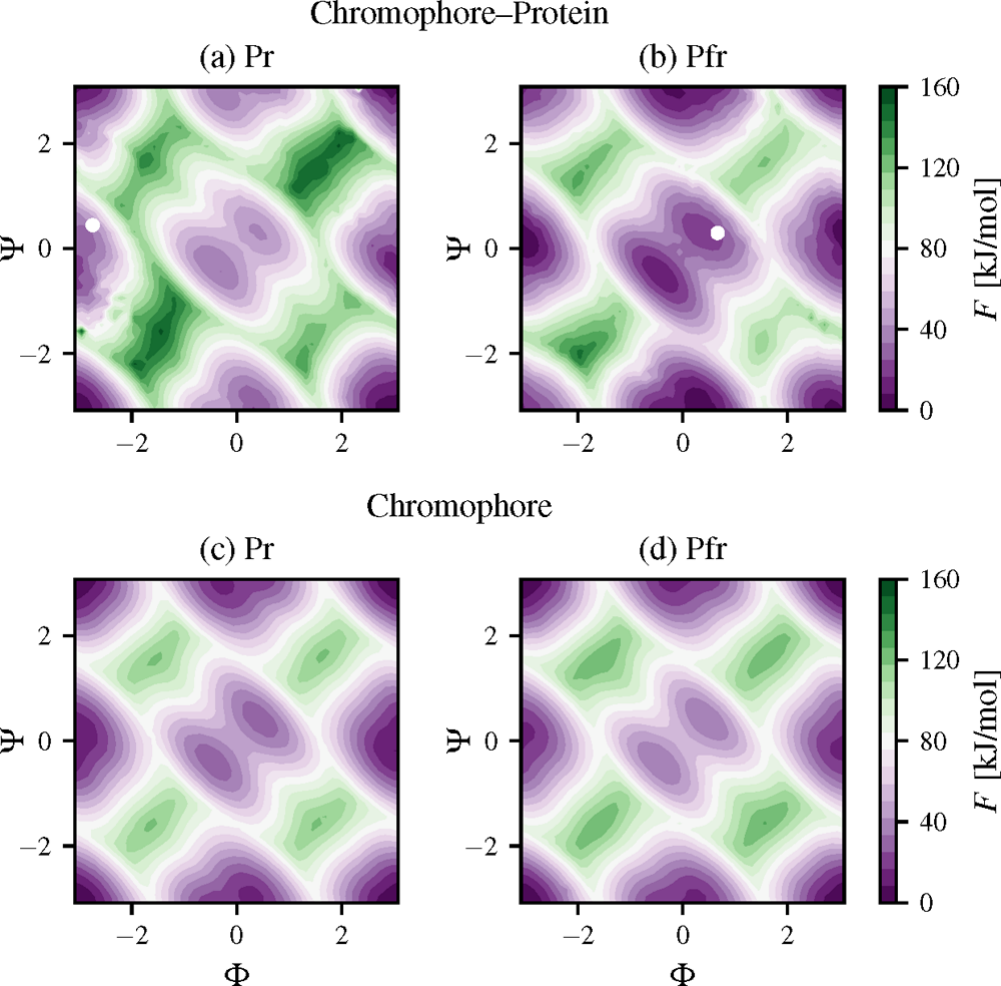
Free energy calculated by biasing the dihedral angles Φ and Ψ (in radians) using variationally enhanced sampling (VES) for different photoactive conformers of BphP-BV: (a) Pr or “red” (PDB: 4O01(17)) and (b) Pfr or “far-red” (PDB: 4O0P(17)). The X-ray structures from the PDB above are indicated by white dots. FE landscapes for the chromophore in solvent are shown in (c) *ZZZ* and (d) *ZZE*.

As typically statistical weights in the initial stages of enhanced sampling simulations are not equilibrated, we empty our simulations of possible sampling artifacts performing a standard preprocessing of the gathered CV samples. Namely, we take into account only the CV samples starting from 10 ns to exclude possibly unreliable samples. We can see that after 10 ns, the weights are more stabilized as is presented in Figures S3 and S4. The remaining samples are used to calculate the FE landscapes. In Figure 2, we show the FE landscapes estimated using the umbrella reweighting.^57^ We calculate also the FE profiles of the systems along the Φ and Ψ dihedral angles by integrating out the unused CVs. As we analyze the difference between global FE minima and maxima in both Pr and Pfr, we place the deepest FE minimum at 0 kJ/mol in each landscape.

To assess the convergence of the enhanced sampling simulations, we check if the simulations efficiently explore the CV space. For BphP-BV, after 500 ns, both simulations cross the CV space multiple times as can be observed from the time-series of the CVs (Figure S5). For the BV chromophore in solvent, the convergence is obtained earlier (Figure S6). At the end of the simulations, the transitions across the CV space are more frequent as the bias potential renders the dynamics close to diffusive. Additionally, to check the relative changes between the FE landscapes of the protein-chromophore complexes in the Pr and Pfr forms, we employ a statistical distance based on the Kullback–Leibler divergence to quantify the averaged difference in the whole FE landscapes calculated at different time steps of the simulations. We can see the distance reaches a plateau at 320 ns (Figure S9a). Additionally, the mean standard deviations of the FE landscapes calculated over the last 200 ns are 1.76 and 0.52 kJ/mol for Pr and Pfr, respectively. Taking the above into consideration, we conclude that the simulations reach the level of convergence required for the further analysis.

**Figure 3:**
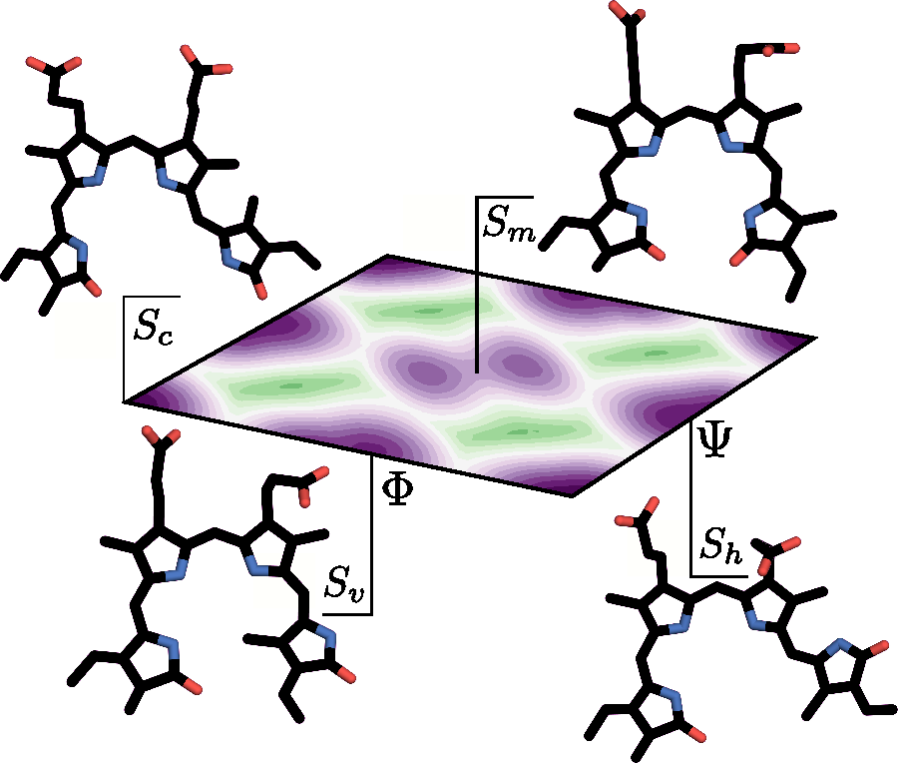
Labels indicating the names of the metastable states used for Pr and Pfr and the respective conformations: corner *S*_*c*_, vertical *S*_*v*_, horizontal *S*_*h*_, and middle *S*_*m*_. The FE landscape is calculated for the BV chromophore in solution. See Figure 2 for further details.

**Figure 4:**
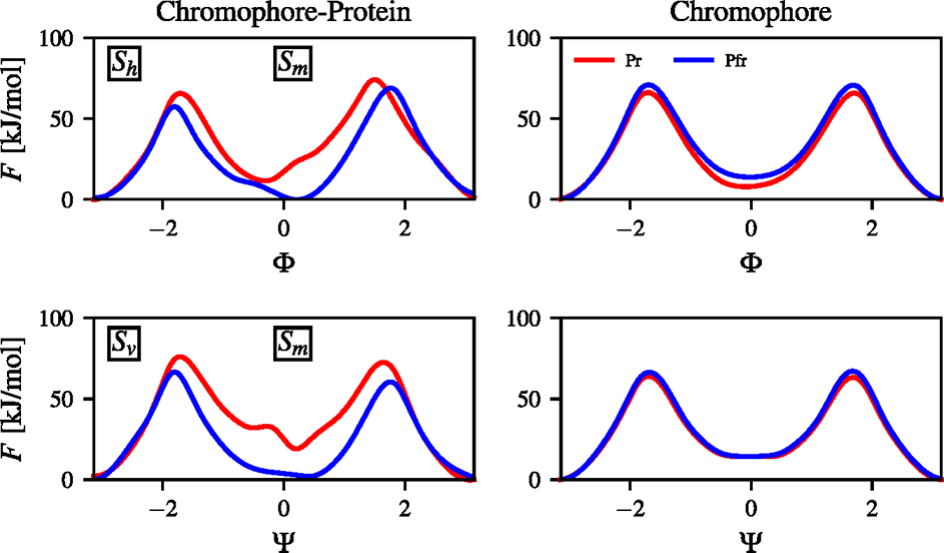
Free energy profiles for the Pr and Pfr conformers of the BphP-BV conformers and the chromophore without the protein environment. For the chromophore–protein the FE profiles are shown in the first column while for the chromophore in the second column. The FE profiles are calculated from the Pr and Pfr FE landscapes in the diherdal angle space (Φ, Ψ) (in radians). Note that the FE profiles provide an averaged description.

We present the FE landscapes associated with biasing the CVs of the Pr in Figure 2a and Pfr in Figure 2b. We also mark the X-ray structures from the PDB files (Pfr, 4O0P, and Pr, 4O01) as white dots. Additionally, we show the FE landscapes of the *ZZZ* (Figure 2a) and *ZZE* (Figure 2b) conformations of the BV chromophore embedded in solvent, i.e., without the protein environment. The FE profiles along each CV are shown in Figure 4. We establish a naming convention that can be seen in Figure 3 where the BV conformations are shown on the FE landscape of the BV chromophore in solution. The metastable states are labeled intuitively: *S*_*m*_ (middle), *S*_*v*_ (vertical), *S*_*h*_ (horizontal), and *S*_*c*_ (corner). The *S*_*h*_ and *S*_*m*_ metastable states are also referred to as the Pr X-ray and Pfr X-ray states, respectively. We label the *S*_*m*_ metastable state as a single superstate rather than two separate states as it consists of two substates between which transitions are frequent enough (Figure 3). As we show and discuss later, this decision is corroborated by our analysis presented in Figure S10.

The FE landscapes of Pr and Pfr consist of four metastable states located in similar CV regions. From parts a and b of Figure 2, we can see that there are distinct differences between the Pr and Pfr FE landscapes in the case of the protein–chromophore complexes. This change in the FE landscapes of the chromophore–protein forms is caused by the different interactions between the BV chromophore and the CBD as shown in the crystallographic structures of Pr and Pfr.^17^ Moreover, the FE landscapes of the solvated chromophore do not show differences between the *ZZZ* and *ZZE* forms. As it can be noticed, the *ZZE* chromophore form, despite initially sampling *S*_*m*_, falls to the *S*_*h*_ metastable state at 0 kJ/mol which is more preferable. Additionally, we can observe that the highest FE barrier of the *ZZZ* and *ZZE* chromophore forms in solvent is around 125 kJ/mol (Figure 2c,d).

The calculated FE barriers are slightly higher for BV in the protein relative to solution. It appears that the protein environment provides interactions that hinder the D-ring rotation. The highest FE barrier for Pr is about 140 kJ/mol, while for Pfr it is about 125 kJ/mol (Figure 2, parts a and b). We can see that the highest FE barriers of the Pfr and Pr forms of the chromophore are very close to that of Pfr in the case of the chromophore–protein system. This fact clearly indicates that the chromophore has more conformational freedom in the CBD in the Pfr form in comparison to the Pr form. This observation can be noticed in Figure S8 where it is visible that the BphP-BV complex in the Pfr form has lower FE barriers than the isolated BV chromophore. From the FE landscapes (Figure 2) and the FE profiles (Figure 4), we can see that both CVs are needed distinguish all distinct metastable states.

### 3.3 Metastable States

We start from the metastable states which contain the X-ray structures of the Pr and Pfr conformers (Figure 2). We can observe that despite lying in the same CV regions, the metastable states of Pr and Pfr are markedly different. As we can see in Figure 2, this difference is indicated by the change in the X-ray metastable states, i.e., states that are populated by the X-ray chromophore conformations. Noticeably, the X-ray state of Pr is slightly shifted with respect to the minimum of the FE basin. For the Pr conformer, *S*_*h*_ is characterized by low FEs. In contrast, the crystallographic state for Pfr is the *S*_*m*_ metastable state. We can see that the change in the X-ray states amounts to rotating the Φ dihedral angle by approximately 180 deg. There is virtually no change in the Ψ dihedral angle if we consider a linear approximation of this transition. Although, as we later discuss, there may be also additional and more complex transition paths that are possible.

Next, we move toward calculating more quantitative differences between the FE landscapes of Pr and Pfr. To this aim, we use a formula for FE differences (Section S8) to compare the FE between the corresponding metastable states in both conformers. Calculating FE differences requires a selection of CV samples from the metastable states. We perform this by first picking only the CV samples belonging primarily to the metastable states using a threshold value to sieve only CV samples with high statistical weights. The remaining samples are negligible as their weight decreases exponentially with the energy according to a Boltzmann factor. Next, we use clustering to group the important CV samples into metastable states. Based on this protocol, we can easily calculate the FE differences between the respective metastable states in Pr and Pfr. We plot these differences in Figure S9b. Additional details regarding the analysis of clustering and the employed protocol can be found in Figure S10 and Figure S11.

We find that there are considerable FE differences between Pr and Pfr (Figure S9b). As we calculate the differences by comparing Pr to Pfr, therefore, a negative value means that a state is more populated in Pr, while a positive value indicates that a state is more populated in Pfr. The highest FE difference is between *S*_*m*_ states, reaching around 25 kJ/mol. This metastable state has a lower FE basin in Pfr than in Pr which can be explained by the fact that *S*_*m*_ is the state to which the Pfr X-ray structure belongs. Slightly lower differences (15–20 kJ/mol) are shown for *S*_*h*_ and *S*_*c*_. This is especially interesting as *S*_*h*_ is the X-ray state of Pfr. The only state more favored for Pr is *S*_*c*_ as it is characterized by lower FE than *S*_*c*_ in Pfr (Figure S9b). These findings corroborate our previous analysis (Figure 2) and further indicate that the differences in the FE landscapes of Pr and Pfr indeed are substantial.

### 3.4 Crossing Free Energy Barriers and Dark Thermal Reversion

In addition to the photoconversion between the Pr and Pfr states, a unidirectional thermal pathway allowing dark reversion from Pfr to Pr is often apparent in canonical phytochromes.^4,30^ Among BV-binding BphPs, only those with a resting Pr state show Pfr structural heterogeneity of the chromophore indicated by a temperature-dependent equilibrium. This is possibly related to the capability of the chromophore to undergo a thermal isomerization and, thus, a reversion to Pr. This dark reversion can be considered a rare event with a specific time scale depending on the type of phytochrome ranging from minutes to hours.^33,59^ Below, we explain how the dark reversion can be rationalized from a thermodynamical point of view.

As previously noted, the crystallographic state of the Pfr form (*S*_*m*_) consists of two substates between which transitions are relatively frequent (Figure 2). The first substate is populated by the X-ray conformation of Pfr. The second substate is separated from the X-ray substate by a low FE barrier (Figure 2b). This fact is in contrast with the *S*_*m*_ state of Pr which is not as populated as its X-ray state (*S*_*h*_). We can see that one possible pathway is joining the X-ray substate of Pfr, going through the intermediate state and the *S*_*h*_ metastable state which is of similar FE to that of the X-ray state of Pr. Such a transition corresponds to the dark thermal reversion from Pfr to Pr. It is more probable than the transition from the X-ray state of Pr to the *S*_*m*_ metastable state as the FE barrier separating these states is much higher for Pr. Additionally, the time scales associated with the thermal reversions are on the order of seconds for the dark thermal reversion of Pfr to Pr and on the order of hours for the thermal reversion from Pr to Pfr (Section S6). This observation suggests that the probability of sampling the thermal reversion in canonical phytochromes is indeed higher for the Pfr to Pr transition. However, for bathy phytochromes, this behavior may be reversed as for such phytochromes the Pfr form is the main resting conformation.^60,61^

**Figure 5:**
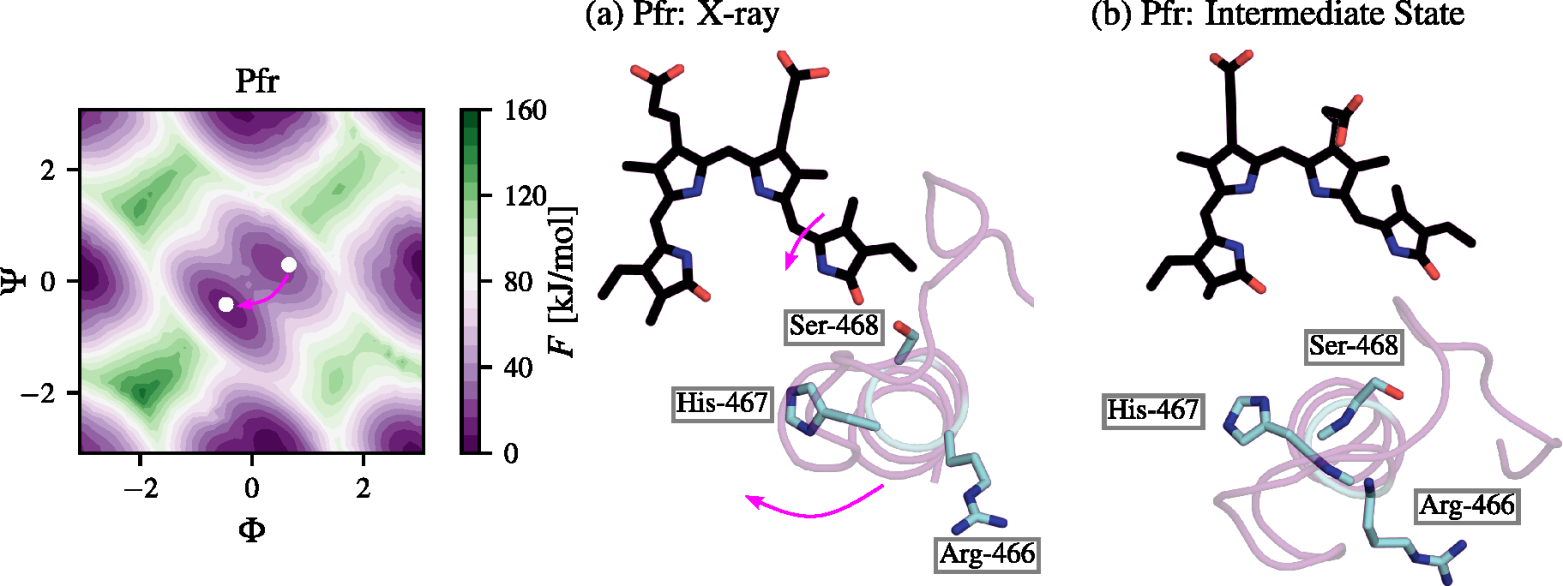
Representative BphP-BV structures of the Pfr conformer in (a) the X-ray FE basin and (b) the intermediate state crucial for the dark thermal reversion toward Pr. The tongue region is shown in magenta and the BV chromophore is shown in black. Amino acids important for the transition from part a to part b are labeled. The dihedral angles Φ and Ψ are shown in radians.

We show the Pfr intermediate state relative to the X-ray state of Pfr in Figure 5. From the presented conformations, we can see that the D-ring of BV in the X-ray Pfr state is stabilized by H-bond to Ser-468. In contrast to this behavior, the intermediate state of Pfr clearly shows that the stabilizing H-bond is broken. This is accompanied by the movement of the tongue region from the position of the BV chromophore (Figure 5b) and a clockwise rotation of Arg-466, His-467, and Ser-468. This fact suggests that the conserved triad in the tongue region has a high impact on the BV chromophore in the CBD and possibly on the α-helix to β-sheet conversion.^62^ The intermediate state seems to be a relevant step in the dark thermal reversion of Pfr to Pr, and indicates that the clockwise rotation of the triad should be more marked in the Pr form. We expand this idea in Section 3.5 and analyze its impact on the secondary structure change of the tongue region and the restriction (Pr) and conformational freedom (Pfr) of the BV chromophore in the CBD.

To explain the changes from Pr to Pfr and from Pfr to Pr, we assume the following transition model on the FE landscape. In the Pr-Pfr transition, the dynamics is started in the X-ray Pr state (*S*_*h*_), is propagated uphill (Figure 2), and crosses the FE barrier of around 100 kJ/mol. Next, the system propagates down the barrier and lands at the FE minimum (30 kJ/mol). Subsequently, the system goes through a series of transient intermediate states^40−44^ (Figure S1) that, due to the limitation of our approach, cannot be detected here and lands at the Pfr FE basin (0 kJ/mol). In the reverse transition (Pfr to Pr), the system starts dynamics in the X-ray FE basin of Pfr (0 kJ/mol), crosses the FE barrier of around 75 kJ/mol, and lands, through Pfr transient conformations, in the *S*_*h*_ metastable state of FE around 0 kJ/mol. In this case, the FE basins are of similar depths (Figure 2a,b).

Using the above assumption, the FE barriers estimated in this study are not in conflict with the absorption spectra of Pr and Pfr. It can be noticed from the above analysis that a higher energy is required in the change from Pr to Pfr in comparison to the change from Pfr to Pr. This can be observed from the FE landscape of Pr in the chromophore–protein case along the Ψ (Figure 2a) as the barrier between the *S*_*h*_ to *S*_*m*_ states of Pr is wider and higher than the barrier between the *S*_*m*_ and *S*_*h*_ of Pfr. Therefore, the Pfr conformer needs lower energy to make such a transition. This result agrees with the nature of the transition between Pr and Pfr. As we stated in the introduction, in the Pr-Pfr transition a red photon triggers the change. In the reverse transition a photon from the far-red spectrum is needed and carries a lower energy than a photon from the red spectrum. Namely, the *Q*-band of the absorption of phytochromes lies within 660–700 nm,^38^ which corresponds to an energy range of around 155–181 kJ/mol. From Figure 2, we can see that the FE barrier of Pr required to pass from *S*_*h*_ to *S*_*m*_ is around 100 kJ/mol, while in the case of Pfr, passing from *S*_*m*_ to *S*_*h*_ is around 75 kJ/mol. These results are also in accord with the fact that the excitation energy must be higher in order to reach an excited state which then converts Pr via the conical intersection to Pfr.^63,64^ Also the energy dissipation in the protein may play a large role^65^ as heat is dissipated to a thermal bath.

### 3.5 Mechanical Signal Transduction

Ligand fluctuation and diffusion are known to drive diverse conformational changes in their protein hosts, as we have previously shown.^66−74^ In the simulations described here, we determine the FE landscape for chromophore isomerization by locally enhancing the chromophore fluctuations, mainly for the D-ring pyrrole of BV. This allows us to test the hypothesis that the mechanical signal propagation in the whole BphP-BV complex can be partially triggered starting with just local BV structural changes. This would then induce changes in the conformations of amino acids in the CBD, and ultimately be propagated to the GAF and PHY domains. As the signal propagates from the CBD to PHY, it is amplified, so that the PHY domain undergoes large conformational changes,^17,75−77^ as shown recently, over time scales longer than explored in our study.

**Figure 6:**
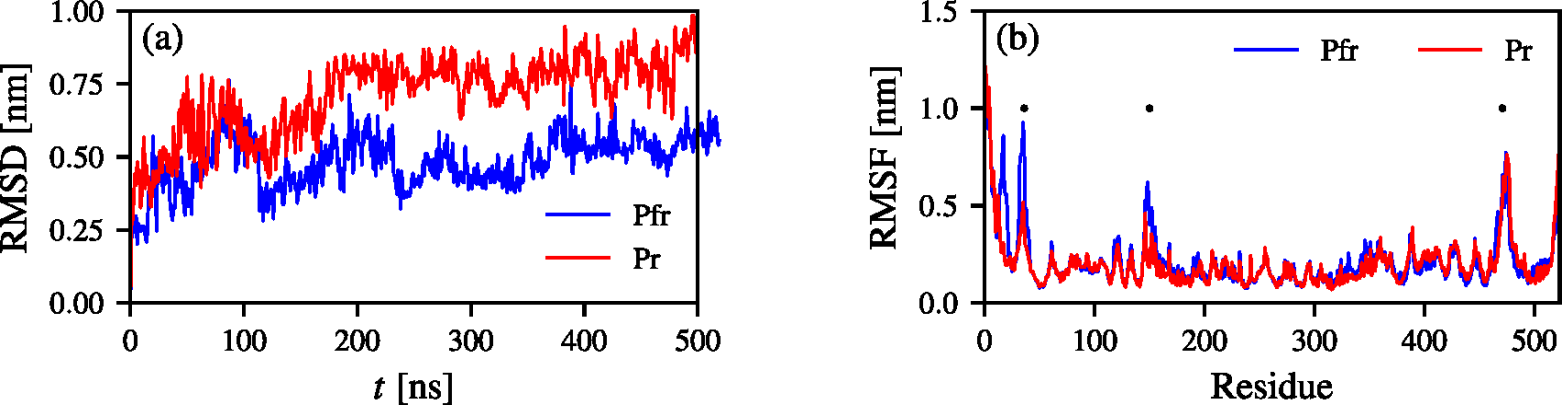
RMS descriptors of the enhanced sampling trajectories. (a) RMSD values calculated by first fitting the C_α_ of the PAS-GAF region to a reference structure. (b) RMSF values calculated for each residue of Pr and Pfr. Dots indicate RMSF values for most fluctuating residue regions. The first curve is the Pr trajectory with a reference structure of the Pr X-ray while the second trajectory (Pfr) is the Pfr trajectory with a reference given by the Pfr X-ray state. For the structures of Pr and Pfr colored by the RMSF values, see Figure S12.

In Figure 6a, we show RMSD values calculated the Pr (Pfr) trajectory obtained using VES in reference to the Pr (Pfr) X-ray state. We can see that the average RMSD values are around 0.5 nm for Pfr and 0.7 nm for Pr. Initially, the enhanced fluctuations of the chromophore D-ring are relatively low, up to around 150 ns, where the RMSD values increase. It is interesting to see that the conformational change in the PHY regions occurs at the same time for the Pr and Pfr conformers. At this point the chromophores in the Pr and Pfr conformers transition through the CV space multiple times (Figure S5), therefore the changes in PHY occur with a considerable delay. In Figure 6b, we show RMSF values for each amino acid of BphP. We can see three distinct peaks indicating enhanced fluctuations in the PAS region (residues 30–70), a small α-helix at the interface of the PAS-GAF domains (residues 120–170), and the tongue region (residues 450–490) in vicinity of the chromophore. The PAS-GAF spine helix suggested in ref (75) to be the main part in the signal transduction in phytochromes is more stable in the Pr form. See Figure S12 for additional results.

Additionally, we analyze the occurrence frequency of residue–residue close contacts (i.e., distance not exceeding 0.35 nm) during the enhanced sampling simulations (Section S11). By identifying the most frequent residue–residue contacts, we can trace where the changes in the structure occur and whether it is possible to link these conformational changes to the enhanced chromophore dynamics. In Figure S12, from these results, we can identify two distinct structural pathways, one proceeding through the partially disordered tongue region and the other associated with the movements of the spine helix.

Taking above into consideration, our results suggest that by enhancing the fluctuations of the BV chromophore in the CBD, the mechanical signal can be indeed partially transferred through the tongue region as shown in ref (17) and the PAS-GAF helical spine to the terminal part of the PHY domain as has been shown by NMR.^75^ Therefore, the subtle effects in the rearrangement in the CBD can also contribute to the overall mechanical signal propagation by both pathways, possibly indicating that the pathways may be biologically relevant and work in tandem.

### 3.6 Interactions in the Chromophore-Binding Domain

To study the interactions between the BV chromophore and the CBD, we calculate the distributions of the number of H-bonds between the chromophore and the protein in Pr and Pfr forms (Figure S13). Our results suggest that, for Pr, the average number of H-bonds between the protein and the chromophore is about twice as large in comparison to Pfr. This indicates that in the Pr conformer the BV chromophore is more constrained in the CBD. In contrast, BV in Pfr can fluctuate more freely (Figure S8). This corroborates our results from calculating the FE landscapes, as the Pfr conformer has FE basins that are wider in comparison to Pr (Figure 2). As the difference in the H-bond distributions is relatively large, it is possible that a collective arrangement of the H-bonds between the chromophore and the photosensory core is a primary driving force that allows for the CBD residues nearest to the chromophore to adjust the protein and affect the Pr and Pfr conformers differently.

From this analysis, we find that the most stabilizing H-bonds for Pr occur between C- and B-rings and Ser-274, Arg-254, Tyr-216, and Ser-257. This is also corroborated by the close contact frequency of residues. For Pfr, the stabilizing H-bonds are almost identical. These propionate side-chain interactions remain intact during the enhanced simulations which suggests a negligible impact of the chromophore photoisomerization in the CBD, in agreement with other studies.^19^

**Figure 7:**
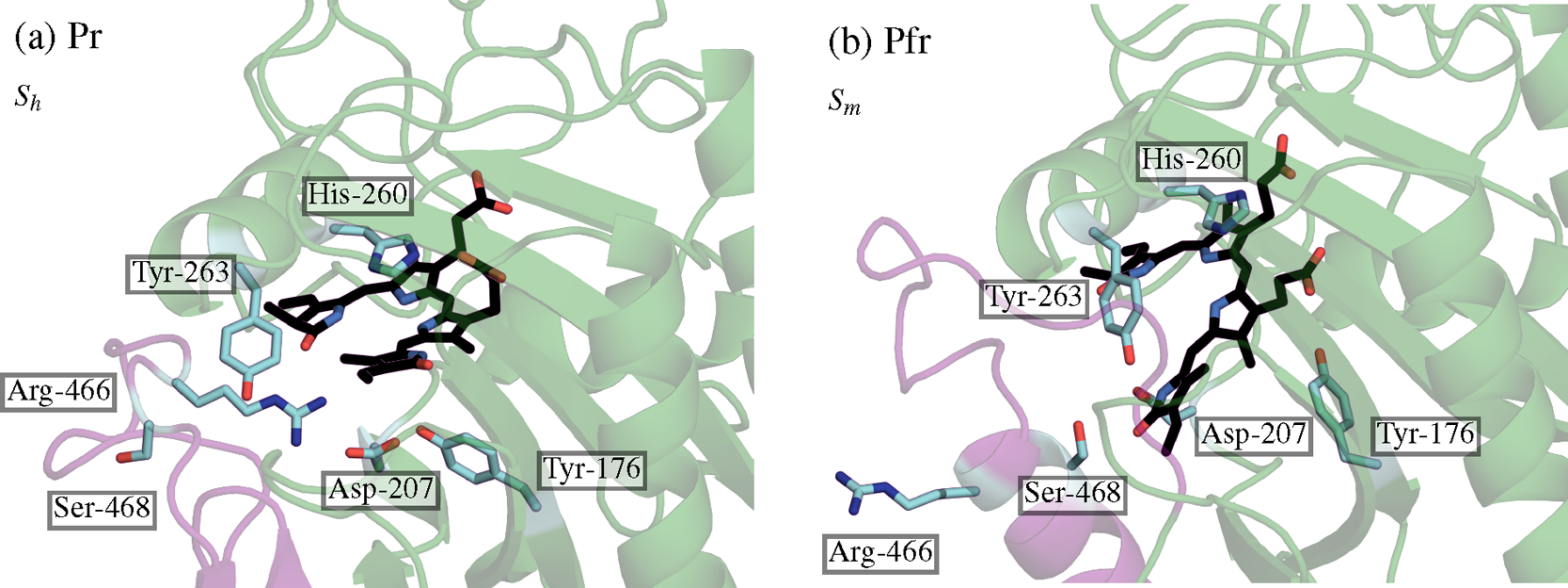
Representative structures of BV-BphP complex taken from the corresponding X-ray metastable basins: (a) Pr, *S*_*h*_, and (b) Pfr, *S*_*m*_. The BV chromophore in the CBD is shown in black, while the labeled amino acids interacting with the D-ring of the chromophore are shown in cyan. The tongue region (residues 450–490) is depicted in magenta while the CBD is shown in green.

In Figure 7, we show the most pronounced changes in the amino acids in the CBD that frequently interact with the BV chromophore. The BV-BphP representative structures are taken from the X-ray metastable basins, i.e., *S*_*h*_ for the Pr conformer (Figure 7a) and *S*_*m*_ for the Pfr conformer (Figure 7). We can see that many amino acids are pushed to the edge of the CBD in Pfr in comparison to Pr, e.g., Tyr-176, Asp-221, and His-260. This structural rearrangement creates more conformational space for the BV chromophore in the Pfr conformer. More importantly, we can observe that the tongue region in Pr (β-sheet) and Pfr (α-helix) is structurally changed in the proximity of BV.

As depicted in Figure 7, in both Pr and Pfr, the OD1 and OD2 atoms of Asp-207 accept the hydrogen from the D-ring nitrogen and the amine group of His-260 stably accepts the hydrogen from the D-ring. The side chain of Asp-207 is a member of a ligand pocket in the CBD. Also Tyr-176 and Tyr-263 belong to this triad located in the proximity of the D-ring. The side chains from the tongue region, Arg-466, His-467, and Ser-468 behave differently in Pr and Pfr. In the Pr conformer, Arg-466 donates two hydrogen atoms to the D-ring nitrogen as a result of highly dynamic H-bonding patters for C=O.^78^ Next, His-467 accepts the H-bond from the D-ring nitrogen. These amino acids stabilize closely the D-ring, constraining its rotation. However, in Pfr, the conformational changes of the tongue region disrupt these interactions by flipping Arg-466 and His-467 outside the pocket (Figure 7b). Then, Ser-468 directs toward the CBD and donates the hydrogen to the D-ring oxygen.

Some discussion is in order regarding to the flip-and-rotate model,^79,80^ according to which the overall in-plane rotation of the BV chromophore relative to the protein environment also plays a role in the rearrangement within the CBD. To observe if this in-plane rotation of BV can be also observed in our simulations, we calculate the mean and error (0.95 confidence interval) values for the time-series of an angle quantifying the rotation for the Pr and Pfr forms. We define this angle based on the CHB and CHC (carbon atoms between the A- and B-rings and B- and C-rings, respectively) of the BV chromophore and the Cα atom of Leu-235 lying in the plane of BV (see Figure S7). We perform this estimation using bootstrapping. Our analysis yields angles of 2.45 ± 0.2 rad and 2.63 ± 0.2 rad for Pr and Pfr, respectively. The BV chromophore exhibits reorientations by about 0.2 rad in both Pr and Pfr states, and there is almost a 0.2 rad change in orientation between two forms, although the means agree within the margin of error. However, as we can see in Figure S7, the distributions of the in-plane angles are different in Pr and Pfr. Namely, the distribution for Pfr is bimodal. The first peak corresponds to the rotations similar to that of Pr (∼2.5 rad) and the second cannot be observed in Pr (∼2.7 rad). The second peak in the distribution of the in-plane angle for Pfr is more populated, and hence, we can observe the propensity of the BV chromophore to assume a conformation different from that in Pr.

Overall, our simulations suggests that the primary photoisomerization event which causes the chromophore to change the rotation angle of the BV D-ring destabilizes the CBD around the D-ring replacing stable interactions to form a loose pocket in which the H-bonds between the CBD and BV are far less frequent. The D-ring rotation makes changes in the disordered part of the tongue which subsequently leads to a secondary structure change of the tongue from an antiparallel β-sheet to an α-helix, but it is the disordered part of the tongue region that allows for that conformational change through direct interactions with the chromophore. Despite the clear differences in the angle distribution of BV in the Pr and Pfr forms, it remains to be further studied if the in-plane rotation of the BV chromophore relative to the protein matrix is an important factor for the rearrangement in the CBD.

## 4 Discussion and Conclusions

We need to underline that, by design, we cannot see the ultrafast photochemical steps during the photoisomerization process in our enhanced sampling MD simulations. However, regardless of the transition through short-lived intermediates,^40−44^ these steps lead the complex to populate the Pfr conformer. What we can see is how different is the conformational behavior of the chromophore within the protein matrix in the Pr and Pfr conformers. By using enhanced sampling MD, we push these conformers out of equilibrium states that are not accessible thermally. Simulating these processes using unbiased MD would only give us a fragment of the whole picture as indicated by recent applications of enhanced sampling MD to photoactive proteins.^74,81^ We plan to employ quantum calculations (e.g., QM/MM) for the photoisomerization process in further studies.

In our work, we discover that the Pr and Pfr conformers are characterized by multiple metastable states. This fact can be uncovered as we monitor and bias both the Φ and Ψ dihedral angles. Conversely, not all metastable states can be observed just by looking at the Φ dihedral angle. This indicates the importance of selecting a set of CVs that can describe the studied systems while losing as little information as possible.^82^ We cannot answer the question if the Φ dihedral is the only required degree of freedom to quantitatively describe the BV-BphP photoisomerization in experimental conditions. However, it is clear that by biasing both dihedral angles, we are able to provide a detailed thermodynamic characterization of Pr and Pfr. Recent studies have shown that photoisomerization events, initially assumed to alter only the double bond rotation, can modify also the single bond.^2,37,83^ From the FE landscapes, we can see that the transition pathways involving both dihedral angles are highly possible, at least in the ground state FE landscapes.

Another open question that cannot be answered even by exploiting the most advanced experimental techniques, is which structural changes of phytochromes are the most important to propagate the mechanical signal from the CBD to the PHY domain. Whereas it is possible to gain general knowledge about the identity of structural rearrangements from IR studies, the insight obtained is far away from an atomic-level resolution. On the other hand, XFMS experiments, which have a single-residue spatial resolution, cannot provide time-resolved data at the required accuracy level. Our model, on the contrary, provides a detailed thermodynamic and atomistic description of the additional driving force caused by the enhancing the local fluctuations of the BV chromophore for the multiscale structural changes of the protein involving the Pr and Pfr conformers. We find that the mechanical signal is possibly transferred through the tongue region^17^ and the PAS-GAF helical spine^75^ to the terminal part of the PHY domain. This observation indicated that the mechanical signal could propagate by pathways found by Takala et al.^17^ via the X-ray structures and by Isaksson et al.^75^ using NMR.

Notably, the FE barriers estimated in this study agree qualitatively with the absorption spectra of Pr and Pfr. We show that for the protein-chromophore, higher energy is required in the change from Pr to Pfr compared to the reverse transition. The *Q*-band of absorption of phytochromes lies within 660–700 nm,^38^ which corresponds to an energy range of around 155–181 kJ/mol. As shown in this study, the estimated FE barriers are in accord with the fact that the excitation energy must be higher to reach an excited state, which converts Pr via the conical intersection to Pfr. We suggest that the energy dissipation in the protein may play a large role^65^ in the difference between the *Q*-band energies and the FE barriers estimated using VES, as some energy in the form of heat is dissipated to a thermal bath.

Perhaps the most important result of our study is a possibility of the dark thermal reversion from Pfr to Pr. It has been shown experimentally that the BV-like chromophore may adopt alternate conformations^84^ which is indicated by the existence of temperature-dependent chromophore conformations in many phytochromes.^35^ The Pfr state of canonical phytochromes has been shown to be heterogeneous in some cases, with a temperature-dependent equilibrium suggested to be important for the thermal reversion.^34^ In this study, we show that an interplay between two different Pfr chromophore conformations exists. These metastable states are separated by a relatively low FE barrier that renders the transitions between those states to be on the microsecond time scale (Section S6). This fact is also in agreement with the study by Salewski et al.,^60^ where it has been suggested that the Pfr conformer is more prone to populating the CBD with heterogeneous transient chromophore conformations.

We show that the disordered part of the tongue region is heavily affected by the rotation of the D-ring pyrrole of BV. Similar behavior has been observed in ligand dissociation.^71^ We also observe that, during the dark thermal reversion, the amino acid triad of Arg-466, His-467, and Ser-468 rotates clockwise, breaking the H-bond between Ser-468 and the D-ring pyrrole. This clockwise rotation seems to be biologically important in the stabilization of the Pr conformer via H-bond with Arg-466 and a closing motion of the CBD around the BV chromophore. This process provides information about how the protein environment affects the heterogeneous photodynamics of the chromophore and the possible conversion between β-sheet to α-helix in the conserved tongue region of BphP.

In summary, this work is a next step toward elucidating the complete mechanism of the photoisomerization of a bilin-like chromophore in phytochromes from the thermodynamical point of view. Considering the agreement with existing experimental data and the consistency of our results, our findings may be important for the basic understanding of the conversion between photoproducts in light-responsive proteins.

## Supporting Information Available

Detailed methods, protocols, and additional figures (PDF)
