# Supplementary material for: Enhancing the Inhomogeneous Photodynamics of Canonical Bacteriophytochrome
Source: J Phys Chem B. 2022 Mar 31;126(14):2647–57. doi: 10.1021/acs.jpcb.2c00131 (PMC9014414; doi:10.1021/acs.jpcb.2c00131)
Supplement: Supplementary file 1 — jp2c00131_si_001.pdf [file jp2c00131_si_001.pdf]

Supporting Information:

Enhancing the Inhomogeneous Photodynamics of Canonical Bacteriophytochrome

Jakub Rydzewski,[*,†] Katarzyna Walczewska-Szewc,[†] Sylwia Czach,[†] Wieslaw Nowak,[†] and Krzysztof Kuczera[‡,¶]

†*Institute of Physics, Faculty of Physics, Astronomy and Informatics, Nicolaus Copernicus University, Grudziadzka 5, 87-100, Torun, Poland*

‡*Department of Molecular Biosciences, University of Kansas, Lawrence, KS 66047, USA*

¶*Department of Chemistry, University of Kansas, Lawrence, KS 66045, USA*

E-mail: jr@fizyka.umk.pl

Contents

S1 Phytochrome Photocycle 3

S2 Equilibrium Metastable States 4

S3 Variationally Enhanced Sampling 4

S4 Time-Series of the CVs 6

S5 Statistical Distance $D_F[F(\mathbf{z}), F'(\mathbf{z})]$ 9

S6 Timescale Estimates 9

S7 Protein Constraint Effect on Free Energy Landscapes 10

S8 Free Energy Differences and Clustering 10

S9 B-Factors 14

S10 Hydrogen Bonds 14

S11 Close Contacts 15

References 15

S1 Phytochrome Photocycle

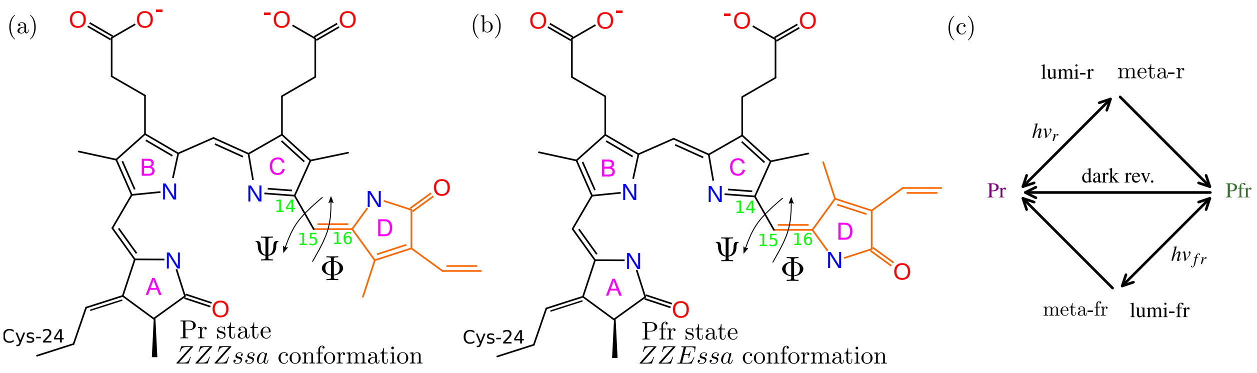

Figure S1: Structures of BV in (a) $ZZZssa$ and (b) $ZZEssa$ conformations belonging to the Pr and Pfr forms, respectively. The BV chromophore is bound covalently to the Cys-24 of BphP. The photoisomerization employs the rotation of the D-ring pyrrole of the BV chromophore (orange). Throughout the simulations performed in this study, we monitor the Φ and Ψ dihedral angles, corresponding to the C15=C16 and C14–C15 bonds (numbers shown in green), respectively. (c) Phytochrome reversible photocycle. Illumination of Pr phytochrome with red light produces lumi-r and meta-r as the primary photoproducts. This is subsequently converted to Pfr. Pfr can be converted into Pr either by illumination with far-red light, producing lumi-fr and meta-fr, and then Pr or via a subsequent thermal process known as the dark thermal reversion known to occur in canonical phytochromes.[1]

Table S1: Protonation of nitrogen atoms in the BV chromophore for Pr and Pfr. Data taken from ref 2.

	NA	NB	NC	ND
Pr	Y	Y	Y	Y
Pfr	Y	Y	Y	Y

S2 Equilibrium Metastable States

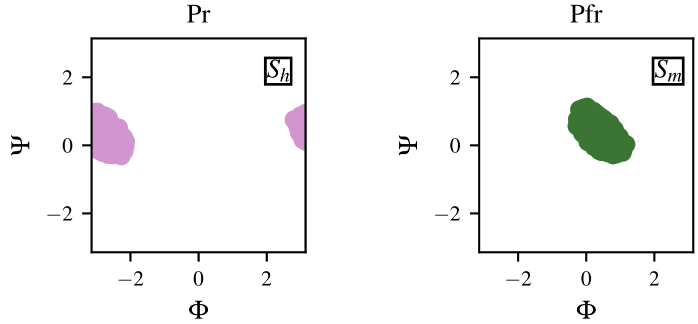

Figure S2: Equilibrium states sampled using 200 ns unbiased molecular dynamics simulations. We can observe that the simulations sample only the metastable states characterized by the X-ray structures. The metastable states are labeled by the names introduced in the main text.

S3 Variationally Enhanced Sampling

Here, we explain the theory behind CV-based enhanced sampling MD methods and a method used in this study to reconstruct FE landscapes. Let us consider a molecular system described by microscopic coordinates, $\mathbf{R}$ and a potential energy function $U(\mathbf{R})$. In the canonical ensemble, $\mathbf{R}$ follows the Boltzmann distribution $P(\mathbf{R}) \propto \mathrm{e}^{-\beta U(\mathbf{R})}$, where β is the inverse of the thermal energy. As molecular systems are high-dimensional ($3N$, where N is the number of atoms), we identify a small set of CVs, $\mathbf{z}(\mathbf{R})$ to reduce the dimensionality. We obtain the marginal equilibrium distribution of $\mathbf{z}$ as:

$$P(\mathbf{z}) = \int \mathrm{d}\mathbf{R}\, \delta[\mathbf{z} - \mathbf{z}(\mathbf{R})] P(\mathbf{R}) = \Big\langle \delta[\mathbf{z} - \mathbf{z}(\mathbf{R})] \Big\rangle_U, \tag{S1}$$

where $\delta[\cdot]$ is the Dirac delta function and $\langle\cdot\rangle_U$ is the ensemble average under the potential $U(\mathbf{R})$.

In systems exhibiting sampling problems (i.e., metastability), the FE landscape, $F(\mathbf{z}) = -\frac{1}{\beta}\log P(\mathbf{z})$, consists of many metastable states separated by energy barriers much larger

than the thermal energy. Therefore, on the timescales we can simulate, the system stays kinetically trapped and is unable to explore the CV space. In such a case the convergence is virtually impossible to reach using standard MD methods.

The sampling problem may be lifted using CV-based enhanced sampling methods where we introduce a bias $V(\mathbf{z})$ acting in the $\mathbf{z}$ space. This idea, however, causes the probability to change from $P(\mathbf{z})$ to:

$$P_V(\mathbf{z}) = \Big\langle \delta[\mathbf{z} - \mathbf{z}(\mathbf{R})] \Big\rangle_{U+V}. \tag{S2}$$

Hence, this trick is called the non-Boltzmann sampling. Over the past decade, several CV-based enhanced sampling methods have emerged, see refs 3–5 and references therein. Any such method can yield to the unbiased probability, $P(\mathbf{z})$, from which we can obtain $F(\mathbf{z})$.

Here, we focus on a recent method devised by Valsson and Parrinello,[6] variationally enhanced sampling (VES). In VES, we optimize a functional dependent on the bias embedded in the CV space $\mathbf{z}$:

$$\Omega[V] = \frac{1}{\beta} \log\left(\frac{\int \mathrm{d}\mathbf{z}\, \mathrm{e}^{-\beta[F(\mathbf{z})+V(\mathbf{z})]}}{\int \mathrm{d}\mathbf{z}\, \mathrm{e}^{-\beta F(\mathbf{z})}} \right) + \int \mathrm{d}\mathbf{z}\, p_T(\mathbf{z}) V(\mathbf{z}) \tag{S3}$$

where $p_T(\mathbf{z})$ is the target distribution toward which the sampling is biased. It follows from eq S3 that up to an immaterial constant, F is given as:

$$F(\mathbf{z}) = -V(\mathbf{z}) - \frac{1}{\beta} \log p_T(\mathbf{z}), \tag{S4}$$

when V is such that $\Omega[V]$ is stationary. If we take p_T as uniform, we have simply $F = -V$. Overall, through the minimization of $\Omega[V]$, we can calculate $F(\mathbf{z})$. Or equivalently, through the standard umbrella-sampling reweighting:[7]

$$F(\mathbf{z}) = -\frac{1}{\beta} \log\Big\langle \mathrm{e}^{\beta V(\mathbf{z})}\, \delta[\mathbf{z} - \mathbf{z}(\mathbf{R})] \Big\rangle, \tag{S5}$$

where the statistical weights associated with $\mathbf{z}$ are given by $\mathrm{e}^{\beta V(\mathbf{z})}$. In VES, $V(\mathbf{z})$ is usually

represented as a linear combination of basis functions.[6]

The functional $\Omega[V_{\boldsymbol{\alpha}}]$ is optimized using averaged stochastic gradient descent[8] with a learning rate of μ. In the optimization, we consider at iteration k the instantaneous parameters $\boldsymbol{\alpha}^k$ and the averaged parameters $\bar{\boldsymbol{\alpha}}^k = k^{-1}\sum_{i=0}^{k}\boldsymbol{\alpha}^i$. The instantaneous parameters are updated according to:

$$\boldsymbol{\alpha}^{k+1} = \boldsymbol{\alpha}^k - \mu\Big[\Omega'(\bar{\boldsymbol{\alpha}}^k) + (\boldsymbol{\alpha}^k - \bar{\boldsymbol{\alpha}}^k)\Omega''(\bar{\boldsymbol{\alpha}}^k)\Big], \tag{S6}$$

where Ω' and Ω'' are the gradient and the Hessian of Ω, respectively. For the interested readers, more details about VES may be found in ref 6.

S4 Time-Series of the CVs

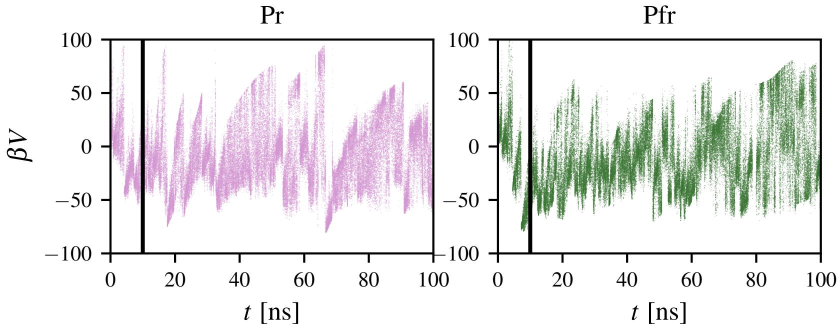

Figure S3: Time-series of the statistical log-weights (βV) for the Pr and Pfr conformers of the protein-chromophore complex during the enhanced sampling simulations. For analysis, we take the samples starting from 10 ns (cut-off indicated by black vertical line).

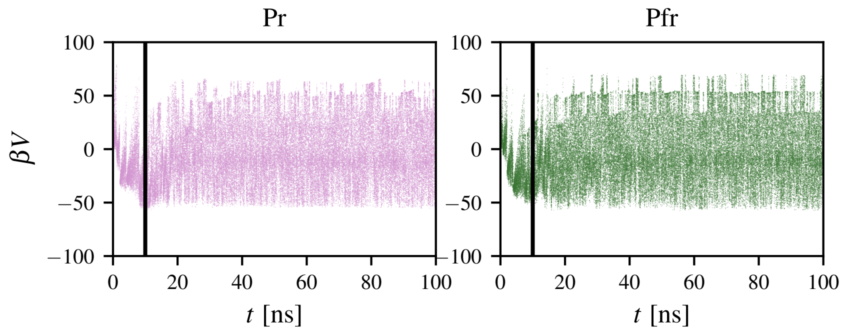

Figure S4: Time-series of the statistical log-weights (βV) for the Pr and Pfr conformers for the chromophore in solvent during the enhanced sampling simulations. For analysis, we take the samples starting from 10 ns (cut-off indicated by black vertical line).

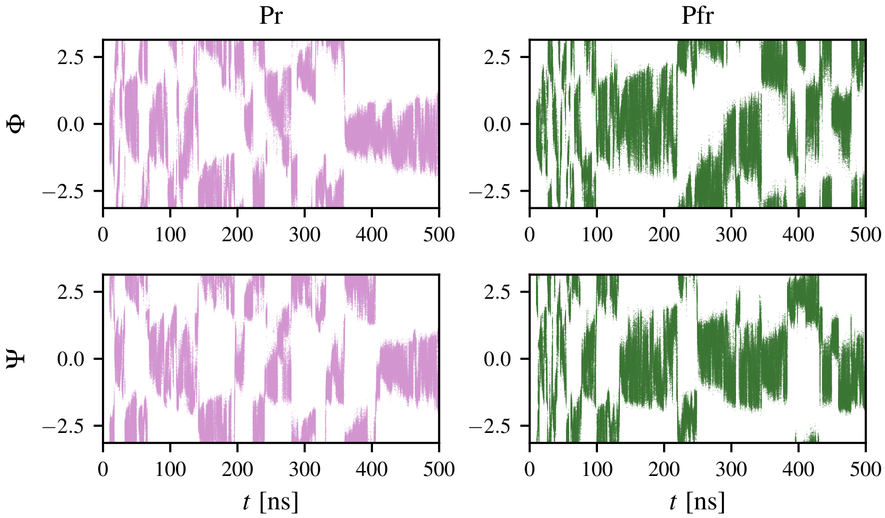

Figure S5: Time-series of the Φ and Ψ CVs for the Pr and Pfr conformers. First 10 ns is not shown due to statistical weights not being equilibrated.

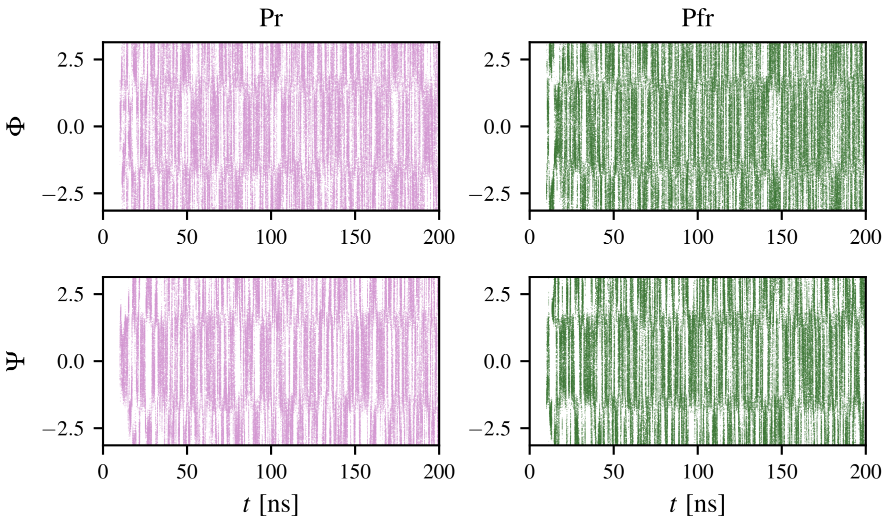

Figure S6: Time-series of the Φ and Ψ CVs for the Pr and Pfr conformers for the chromophore in solvent.

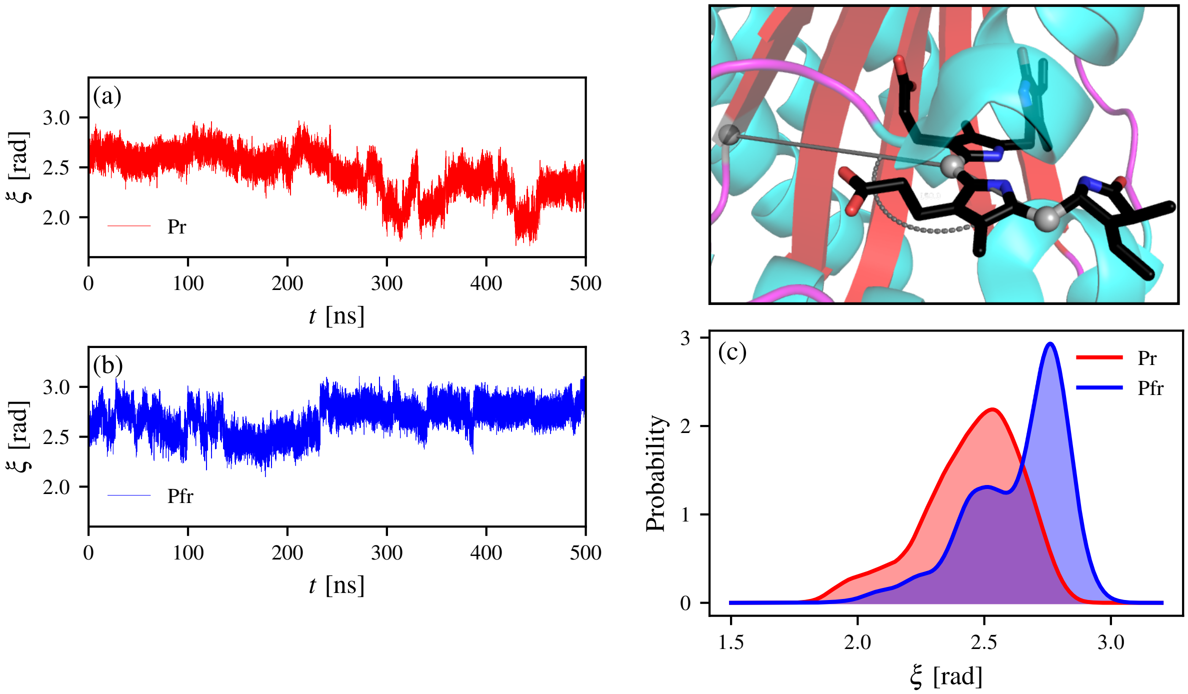

Figure S7: Time-series of the in-plane rotation angle ξ of the BV chromophore relative to the protein defined for CHB, CHC (BV), and Cα of Leu-235. (a-b) The time-series for Pr and Pfr are shown in red and blue, respectively. (c) Histograms of the in-plane angle for Pr and Pfr.

S5 Statistical Distance $D_F[F(\mathbf{z}), F'(\mathbf{z})]$

Here, we introduce a statistical metric based on the Kullback-Leibler divergence that estimates the difference between two FE landscapes. A statistical distance between probability distributions $P(\mathbf{z})$ and $P'(\mathbf{z})$ can be measured using many divergences. Here, we start our derivation from the Kullback-Leibler divergence:

$$D_{\mathrm{KL}}\big[P(\mathbf{z}), P'(\mathbf{z})\big] = \int \mathrm{d}\mathbf{z}\, P(\mathbf{z}) \log\left(\frac{P(\mathbf{z})}{P'(\mathbf{z})}\right), \tag{S7}$$

where $D_{\mathrm{KL}}[P(\mathbf{z}), P'(\mathbf{z})] = 0$ only if $P(\mathbf{z}) \equiv P'(\mathbf{z})$. Next, we use the basic expression for FE, $F(\mathbf{z}) = -\beta^{-1} \log P(\mathbf{z})$, and use it to change the variables in eq S7:

$$D_{\mathrm{KL}}[F(\mathbf{z}), F'(\mathbf{z})] = -\beta Z^{-1} \int \mathrm{d}\mathbf{z}\, \mathrm{e}^{-\beta F(\mathbf{z})} \big[F(\mathbf{z}) - F'(\mathbf{z})\big], \tag{S8}$$

where the partition function is $Z = \int \mathrm{d}\mathbf{z}\, \mathrm{e}^{-\beta F(\mathbf{z})}$. As Z is constant, we can remove it from eq S8. It follows that:

$$D_{\mathrm{KL}}[F(\mathbf{z}), F'(\mathbf{z})] \propto -\beta \int \mathrm{d}\mathbf{z}\, \mathrm{e}^{-\beta F(\mathbf{z})} \big[F(\mathbf{z}) - F'(\mathbf{z})\big] = D_F[F(\mathbf{z}), F'(\mathbf{z})], \tag{S9}$$

where D_F is the divergence used in the main text to calculate differences between the FE landscapes in $\mathbf{z}$ (eq S7) at consecutive time points in our simulations. We can interpret eq S9 as an averaged pairwise difference between $F(\mathbf{z})$ and $F'(\mathbf{z})$ weighted by a factor $-\beta\, \mathrm{e}^{-\beta F(\mathbf{z})}$ which we can interpret as $\propto -\beta P(\mathbf{z})$.

S6 Timescale Estimates

To estimate the order of timescales involved in the dark thermal reversion, we use the Eyring equation:

$$k = \frac{\kappa}{\beta h}\, \mathrm{e}^{-\beta F^{\ddagger}}, \tag{S10}$$

where k is the reaction rate, κ is the transmission coefficient, h is Planck's constant, β is the inverse of thermal energy, and $F^{\ddagger}$ is the height of an FE barrier. We take κ equal to 1. The half-life time can be computed from $t = \log 2/k$.

Considering the Pr conformer, the transition from its X-ray state (S_h) to S_m requires overcoming an FE barrier of about 100 kJ/mol which occurs on the order of hours ($k \sim 2.43 \times 10^{-5}$ s^{-1}). For the Pfr conformer, the system can be seen as a mixture of thermally-dependent X-ray state and the intermediate as transitions between them occur on the order of microseconds. To move to the S_h metastable state, Pfr must overcome an FE barrier of about 75 kJ/mol which amounts to an estimated timescale of seconds ($k \sim 0.55$ s^{-1}).

S7 Protein Constraint Effect on Free Energy Landscapes

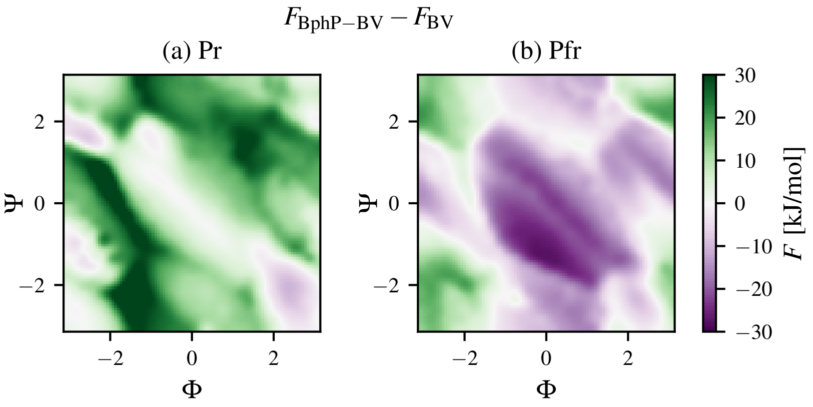

Figure S8: Protein constraint effect on FE landscapes of the Pr and Pfr conformers estimated by calculating differences between FE landscapes of the protein-chromophore complex and the isolated chromophore.

S8 Free Energy Differences and Clustering

We can calculate FE differences by integrating over the metastable aggregate probabilities:

$$\Delta F = -\frac{1}{\beta} \log\left(\frac{\int_k \mathrm{d}\mathbf{z}\, \mathrm{e}^{-\beta F(\mathbf{z})}}{\int_l \mathrm{d}\mathbf{z}\, \mathrm{e}^{-\beta F(\mathbf{z})}}\right), \tag{S11}$$

where the integration domains are the regions in the CV space corresponding to the metastable states k and l, respectively. A clustering of conformations required to calculate FE differences (eq S11) is performed using variational Bayesian estimation of a Gaussian mixture as implemented in the `scikit-learn` library.[9] Each component in the mixture has its own covariance matrix. The weights, means and covariances are initialized using the k-means clustering. The number of components in the Gaussian mixture (e.g., number of clusters) is determined calculating the average silhouette coefficient as shown in Figure S11.

Overall, we use the following workflow to estimate the FE differences of the metastable states in Pr and Pfr:

1. Create a histogram of CV samples and convert it to FE landscape using $F(\mathbf{z}) = -\beta^{-1} \log P(\mathbf{z})$. The histogram must be reweighted with statistical weights $w(\mathbf{z}) = \mathrm{e}^{\beta V(\mathbf{z})}$.

2. Sieve out CV samples above a threshold value (here 70 kJ/mol) to prepare a training data set for clustering. CV samples has negligible value of $w(\mathbf{z}) = \mathrm{e}^{\beta V(\mathbf{z})}$.

3. Cluster (any clustering method may be used) the remaining CV samples for a range of different numbers of clusters and use silhouette analysis to find an optimal number of clusters.

4. Calculate the FE differences between the pairs of metastable states using eq S11.

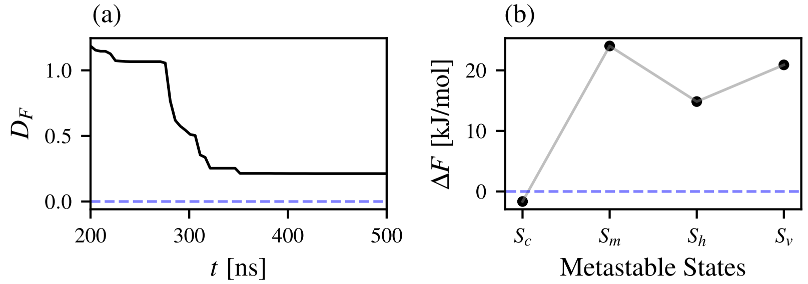

Figure S9: (a) Statistical distance, $D_F[F(\mathbf{z}), F'(\mathbf{z})]$, used to compare the FE landscapes at a global scale. Here, we take the FE landscape of Pr and Pfr as $F(\mathbf{z})$ and $F'(\mathbf{z})$, respectively. The FE landscapes are calculated by using samples up to time t. (b) FE differences between the corresponding metastable states in Pr and Pfr using eq S11. The x axis shows the names of the metastable states.

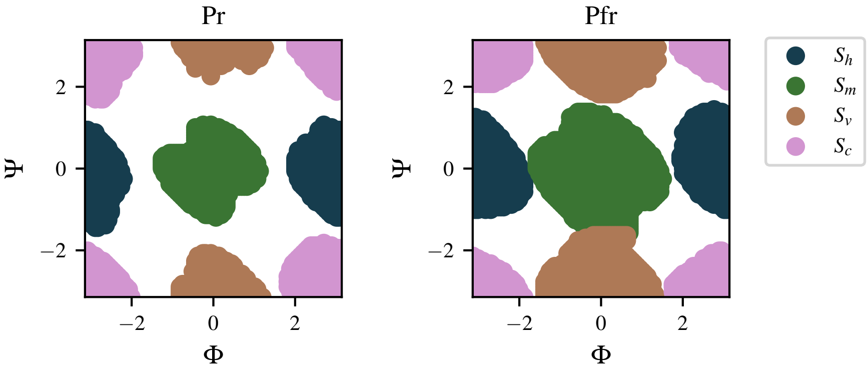

Figure S10: Clustering results of variational Bayesian Gaussian mixture calculated for the number of components (clusters) set to 4 as indicated by the silhouette analysis (Figure S11). The clustered metastable states of Pr and Pfr are shown in different colors. The naming convention as in the legend is used in the main text.

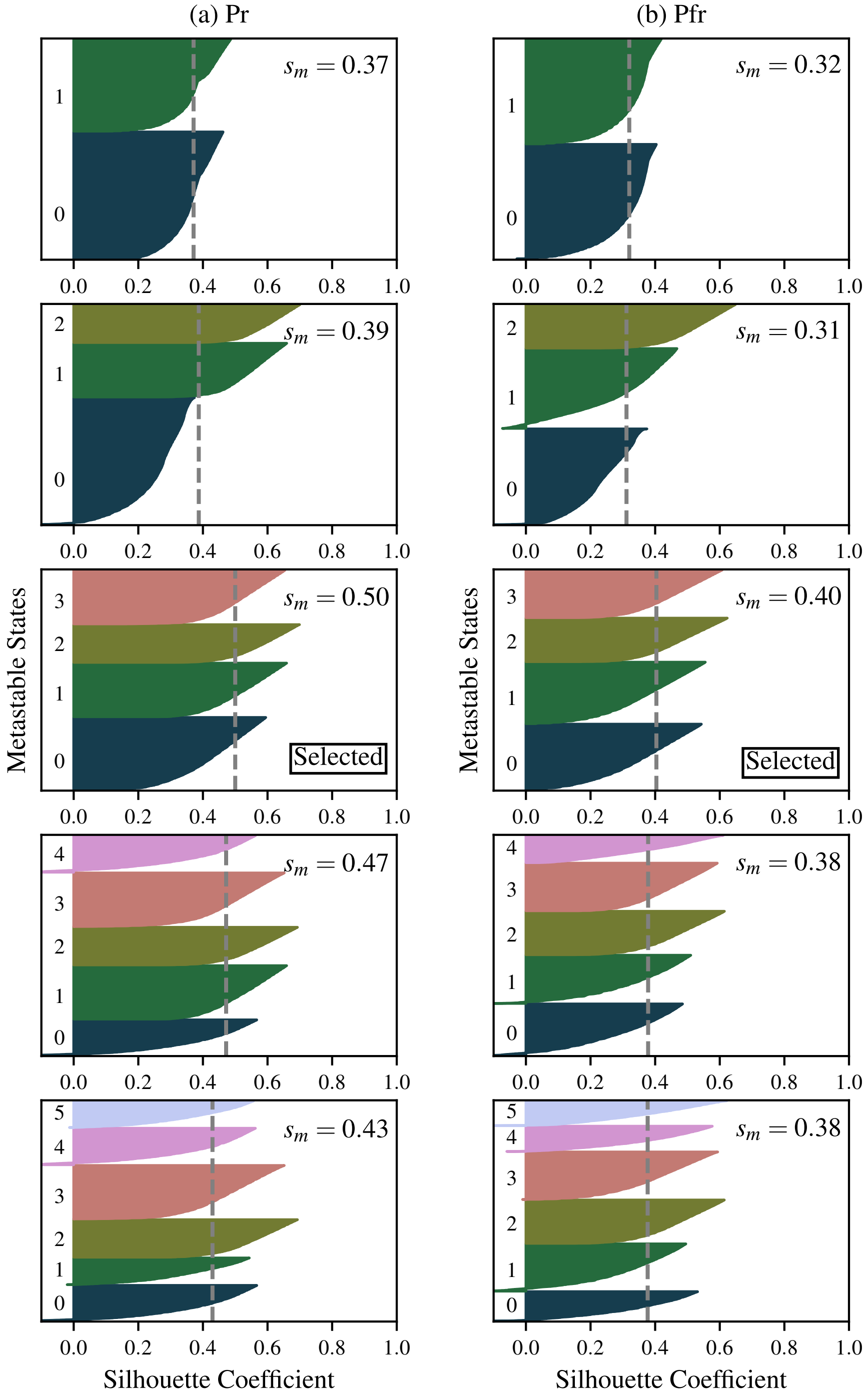

Figure S11: Selecting the number of components with silhouette analysis using Gaussian mixture clustering for (a) Pr and (b) Pfr. The mean silhouette score is denoted as s_m. The silhouette score has a value from -1 to $+1$. A value near $+1$ indicates that the sample is far away from clusters; a value of 0 that the sample is on or close the edge of the neighboring clusters; negative values show an error in assignment.

S9 B-Factors

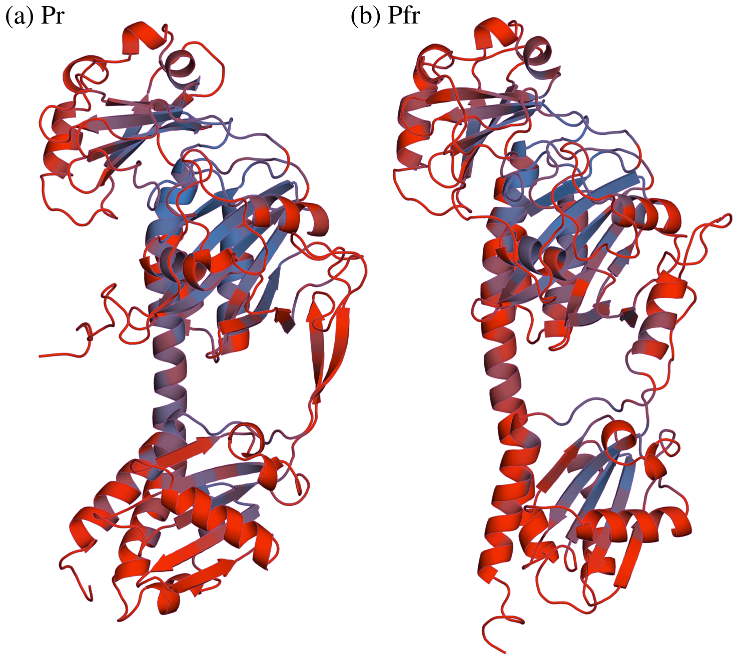

Figure S12: Structures of (a) Pr and (b) Pfr with residues colored by B-factor values calculated from RMSF. The main difference lies in the PAS-GAF helix spine that is more flexible in the Pfr conformer than in the Pr conformer.

S10 Hydrogen Bonds

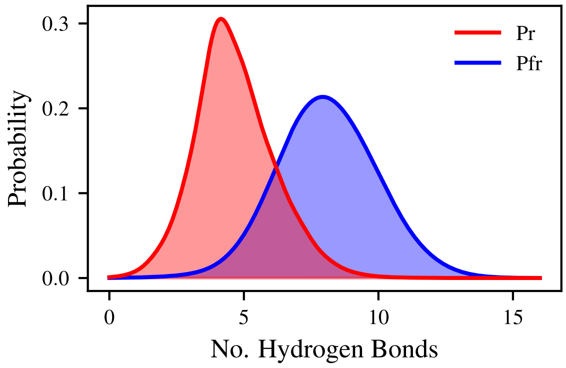

Figure S13: Histogram of the number of hydrogen bonds during the VES simulations for the Pr and Pfr conformers. Hydrogen bonds are calculated between the BV chromophore and the BphP protein.

S11 Close Contacts

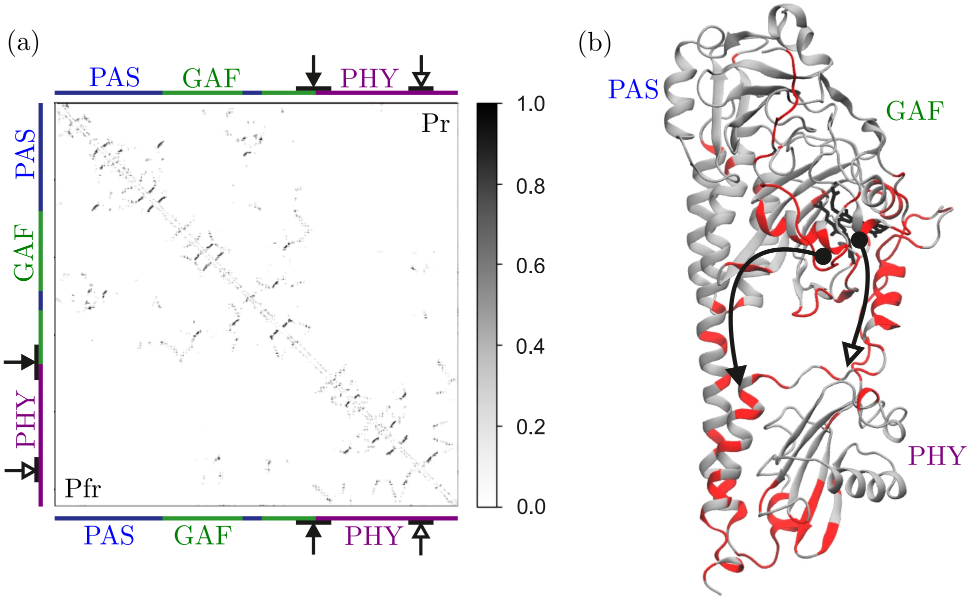

Figure S14: Pairwise close contact occurrence during the enhanced sampling simulations for the Pr and Pfr conformers. (a) Black and white-headed arrows indicate regions of the tongue region and the helix spine. Putative signal transduction pathways are shown in red as the regions with the greatest difference (> 0.7) in pairwise contact frequency between the Pr and Pfr models.

References

(1) Quail, P. H. Phytochrome Photosensory Signalling Networks. *Nat. Rev. Mol. Cell Biol.* **2002**, *3*, 85–93.

(2) Modi, V.; Donnini, S.; Groenhof, G.; Morozov, D. Protonation of the Biliverdin IXα Chromophore in the Red and Far-Red Photoactive States of a Bacteriophytochrome. *J. Phys. Chem. B* **2019**, *123*, 2325–2334.

(3) Abrams, C.; Bussi, G. Enhanced Sampling in Molecular Dynamics using Metadynamics, Replica-Exchange, and Temperature-Acceleration. *Entropy* **2014**, *16*, 163–199.

(4) Valsson, O.; Tiwary, P.; Parrinello, M. Enhancing Important Fluctuations: Rare Events

and Metadynamics from a Conceptual Viewpoint. *Ann. Rev. Phys. Chem.* **2016**, *67*, 159–184.

(5) Bussi, G.; Laio, A. Using Metadynamics to Explore Complex Free-Energy Landscapes. *Nat. Rev. Phys.* **2020**, 1.

(6) Valsson, O.; Parrinello, M. Variational Approach to Enhanced Sampling and Free Energy Calculations. *Phys. Rev. Lett.* **2014**, *113*, 090601.

(7) Torrie, G. M.; Valleau, J. P. Nonphysical Sampling Distributions in Monte Carlo Free-Energy Estimation: Umbrella Sampling. *J. Comp. Phys.* **1977**, *23*, 187–199.

(8) Bach, F.; Moulines, E. Non-Strongly-Convex Smooth Stochastic Approximation with Convergence Rate $O(1/n)$. *NeurIPS* **2013**, *26*, 773–781.

(9) Pedregosa, F.; Varoquaux, G.; Gramfort, A.; Michel, V.; Thirion, B.; Grisel, O.; Blondel, M.; Prettenhofer, P.; Weiss, R.; Dubourg, V.; Vanderplas, J.; Passos, A.; Cournapeau, D.; Brucher, M.; Perrot, M.; Duchesnay, E. `scikit-learn`: Machine Learning in Python. *J. Mach. Lear. Res.* **2011**, *12*, 2825–2830, http://jmlr.org/papers/v12/pedregosa11a.html.
